# State Medical Examinations

**Published:** 1896-02

**Authors:** William Warren Potter

**Affiliations:** Buffalo, N. Y.; Member New York State Medical Examining and Licensing Board. President National Confederation State Medical Examining and Licensing Boards, 1896


### STATE MEDICAL EXAMINATIONS.

CONDUCTED BY WILLIAM WARREN POTTER, M. D., Buffalo, N. Y.
Member New York State Medical Examining and Licensing Board.
President National Confederation State Medical Examining and Licensing Boards, 1896.

### UNIVERSITY OF THE STATE OF NEW YORK MEDICAL EXAMINATIONS.

EXAMINATIONS for license to practise medicine in this state will be held as follows :

*Dates.*—1896 : January 28–31, April 7–10, May 19–22, June 16–19.

*Places.*—New York, Albany, Syracuse, Buffalo. Each candidate is notified as to exact place.

*Daily Program.*—Tuesday, morning, 9.15—12.15, anatomy ; afternoon, 1.15—4.15, physiology and hygiene. Wednesday, morning, 9.15—12.15, chemistry ; afternoon, 1.15—4.15, surgery. Thursday, morning, 9.15—12.15, obstetrics ; afternoon, 1.15—4.15, pathology and diagnosis. Friday, morning, 9.15—12.15, therapeutics.

---

### PROVISIONAL EXECUTIVE COUNCIL.

THE president of the National Confederation of State Medical Examining and Licensing Boards, Dr. William Warren Potter, of Buffalo, has made the following appointments :

To be members of the Provisional Executive Council :

Dr. Hugh M. Taylor, Richmond, Va.

Dr. Perry H. Millard, St. Paul, Minn.

Dr. Joseph M. Mathews, Louisville, Ky.

Dr. William S. Foster, Pittsburgh, Pa.

These men are well known as earnest advocates of reform in methods of medical education and their appointment gives promise of activity on the part of the national organisation. Dr. Taylor was for years president of the Virginia State Medical Examining Board. Dr. Millard held a similar place in the Minnesota Board, and both of these gentlemen were instrumental in framing legislation and administering laws in their respective states that may be classed as models in regard to the practice of medicine. Dr. Mathews is now president of the Kentucky State Board and has been instrumental in bringing about great reforms in the blue grass state, while Dr. Foster, as secretary and executive officer of the Pennsylvania Board, brings a wealth of experience to the Council from the keystone state.

MEDICAL STUDENT CERTIFICATES.

The regents of the University of the State of New York, on November 21, 1895, voted that the examination committee make a report on the requirements in point of preliminary education for medical degrees. After this meeting an amendment to laws of 1895, chap. 661, was drafted to meet, as far as possible, criticisms and suggestions received at that date. This bill was put before a meeting of the executive committee of the regents, held December 17th, and referred with their approval to the legislative committees of the state medical societies. In order, however, to give a better opportunity for suggestions, a copy of these proposed amendments was sent to each medical school and to the men most interested in the provisions for the licensing of physicians. As a result additional suggestions were received, which led to further changes and a second draft was sent out January 2, 1896, embodying these suggestions.

The examination committee has sent out a third draft, revised to meet as far as possible all the criticisms and suggestions which have been offered.

Following is a synopsis of the amendments:

1. Applicants for admission to the licensing examination must have studied medicine not less than three full school years of not less than nine months each, including three satisfactory courses of at least six months each in three different calendar years.

This will permit students to graduate at the close of the third school year without being compelled to wait, as the present law provides, till the expiration of full thirty-six months.

Six months in each year is the minimum term of instruction in the medical school. The law allows a vacation of three months in each year, but each student is to spend the remaining three months in study, either in a medical school or under a preceptor, or in private.

2. New York medical schools and New York medical students shall not be discriminated against by the registration of any medical school out of the state whose minimum graduation standard is less than that fixed by statute for New York medical schools.

3. A medical school may matriculate conditionally a student deficient in one or more subjects of the preliminary education requirement, provided the deficiency be made up before the student begins the second annual medical course counted toward the degree.

This will enable students to make up entrance conditions and will enable those who come from out of the state for a second course of lectures in New York to meet the preliminary requirement before beginning the second annual medical course counted toward the degree.

4. Students may be allowed to graduate under the conditions which prevailed at the time they began the prescribed study of medicine, without limit as to date of graduation.

5. All medical student certificates granted before this act takes effect may be accepted without limitation as to date of graduation as meeting the preliminary education requirement for degrees.

6. All matriculants after January 1, 1897, must secure forty-eight academic counts or their full equivalent before beginning the first annual medical course counted toward the degree, unless conditionally admitted, when the deficiency must be made up before the student begins the second annual medical course counted toward the degree.

It is considered of great importance that a bill embodying these measures should be introduced in the legislature without delay. A meeting was held at the regents' office at Albany, January 22, 1896, to which all interested were invited, for final consideration of the bill. It now goes to the legislative committees of the state medical societies who have charge of the introduction and conduct of the bill through the legislature.

---

### ENTRANCE REQUIREMENTS OF MISSOURI MEDICAL COLLEGES.

It is announced (*Medical Review*, October 5, 1895,) with much satisfaction that among the minimum requirements of the State Board of Health of Missouri for entering a medical college, recognised by said board, is one which demands the presentation of a diploma of graduation from a good literary and scientific college or high school, or a first grade teacher's certificate. We are also pleased to note that "a college in good standing with the board shall be one in which (other qualifications existing) the course of instruction is a graded one."

By the action of the Missouri State Board of Health a reform in medical education in that state has been inaugurated which has again and again been pointed out and recommended by the best men in the profession, and by men in whom the motive of selfishness is entirely out of the question. The profession, as well as the public in general, will be greatly benefited by the new law.

---

### ENFORCE THE LAW.

OUR medical bill (*Atlanta, Ga., Clinic*,) has been in operation now for some months, and its beneficial results are evident even at this early period, but some "*advertising specialists*" are evidently evading its provisions and engaging in their deceptive practices in our midst. The so-called christian scientists, faith healers, hypnotists, etc., of whom this city is now filled, clearly come under the pale of this recent legislation, and should be made to conform rigidly to all the requirements imposed therein. Look after these intruders and make examples of a few of the more prominent ones, and the more insignificant will be held in abeyance and soon the community rid of such nuisances.

---

### THE OREGON MEDICAL PRACTICE ACT.

THE law regulating medical practice in Oregon, lately passed, seems to be meeting with some difficulties in its enforcement. The *Medical Sentinel* cites two cases where justice miscarried. One case, that of a Chinaman, who was practising without a license in Portland, was tried three times before conviction could be obtained, and then he was released on payment of a fine of $50. Another case was that of a physician who was arrested for practising in Salem without a license, and he was tried and acquitted.

The difficulty seems to be, as the *Sentinel* truly says, that the people do not understand that these laws are for their own protection, and not for that of physicians. The *Sentinel* concludes a very timely editorial on this subject, as follows:

Every licensed practitioner in the state, as an individual, and the state medical society, as a body, should bring all their influence to bear on having the law complied with, or else find out the reason why it is not.

---

### PRACTICE LAWS IN NEW ENGLAND.

ACCORDING to Dr. Charles McIntire (*Atlantic Medical Weekly*,) there are but thirteen states (including territories) whose laws at the present time practically permit any one to practise medicine within their borders, and of these New England claims but one,—New Hampshire.

Four states merely exercise a supervision of the diploma held by the person desiring to practise.

Fourteen require an examination, but accept as an equivalent the diploma of certain medical colleges, and of these New England claims Connecticut, Massachusetts, Rhode Island and Vermont.

Maine alone is in the front rank with those states which require in all cases an examination before a licensing board.

---

### AN UNJUST CRITICISM.

The smart young man who sends "Philadelphia Notes" to the *Journal of the American Medical Association* is responsible for the following attempt to smirch the work of the three-board system in Pennsylvania :

This is the way the results of the work of the state board of Medical Examiners and Licensers appear in the public prints: "The three state medical boards have sent in reports for the last examinations which they conducted. They are: 'Allopathic' Board, 76 examined, 22 failed ; 'Homeopathic' Board, 14 examined, 8 failed ; 'Eclectic' Board, 1 examined, none failed."—*Public Ledger*, Jan. 7, 1896.

It would certainly appear that the praiseworthy effort to elevate medical standards has thus far resulted in advancing the cause of sectarian medicine and placing scientific practitioners on a par with homeopaths and eclectics. Who are "allopathic" physicians, anyhow ?

There are some persons who cannot yet understand that in most of the states the statutes recognise three so-called schools of medicine, namely, one represented by the medical society of the state of ———, one represented by the homeopathic medical society of the state of ———, and one represented by the eclectic medical society of the state of ———.

In order to obtain legislation establishing separate state medical examining and licensing boards in such states, it is necessary to recognise this principle or go without the much-needed reform.

After all, it seems to us that it matters little about the name under which good is accomplished. The fact remains that in the present instance out of ninety-one candidates examined by the three boards, thirty were rejected as incompetent to practise medicine—a long stride toward placing the science of medicine on a sound footing, no matter under what name it was done, and a sufficient answer to the aspersion attempted in the "note" quoted.

